# A study of correlations between metabolic syndrome factors and osteosarcopenic adiposity

**DOI:** 10.1186/s12902-021-00880-w

**Published:** 2021-10-29

**Authors:** Yu-Hsiang Su, Yu-Ming Chang, Chih-Ying Kung, Chiu-Kuei Sung, Wei-Shin Foo, Mei-Hua Wu, Shang-Jyh Chiou

**Affiliations:** 1 Department of Health Care Management, National Taipei University of Nursing and Health Sciences, Taipei, Taiwan; 2 Department of Urology, West Garden Hospital, Taipei, Taiwan; 3 Department of Medical Research and Education, West Garden Hospital, Taipei, Taiwan; 4 Department of Nursing, West Garden Hospital, Taipei, Taiwan; 5 Department of Rehabilitation, West Garden Hospital, Taipei, Taiwan; 6 Institute of Health Policy and Management, College of Public Health, National Taiwan University, Taipei, Taiwan

**Keywords:** Metabolic syndrome, Osteosarcopenic adiposity, Multinomial logistic regression

## Abstract

**Background:**

Aging reduces the quality and strength of bones and muscles and increases body fat, which can lead to the simultaneous occurrence of sarcopenia, osteopenia, and adiposity, a condition referred to as OsteoSarcopenic Adiposity (OSA).

While previous studies have demonstrated that metabolic syndrome is associated with sarcopenia, osteopenia, and adiposity, the relationship between metabolic syndrome and OSA remains largely unknown.

**Methods:**

We analyzed data for a sample of middle-aged individuals from a Health Management Center database, which was collected in 2016–2018. There are 2991 cases of people over 50 years from a physical examination center in a hospital in Taiwan during 2016–2018. In addition to descriptive statistics, chi-squared test, analysis of variance, and multinomial logistic regression analysis were conducted to examine OSA risk and associated factors.

**Results:**

Based on multinomial logistic regression analysis, in different OSA severity level (1–3 more serious), those who are with metabolic syndrome has increased the 2.49–2.57 times risk of OSA (*p* < 0.001) in OSA = 2 and 3 groups while there is no significant difference in OSA =1 group.

**Conclusion:**

The prevalence of OSA may impair the health and quality of life in the elderly group, especially those diagnosed with metabolic syndrome, increasing the risk of OSA. These results can help promote early diagnosis and treatment of OSA in clinical settings, particularly among aging individuals with abnormal physical function, the group with the highest OSA incidence.

## Introduction

While the life expectancy in Taiwan is approximately 81 years, the healthy life expectancy is only 71 years [1]. Generally, this 10-year gap is caused by diseases and loss of physical function that hinder independent living [2]. Therefore, it is especially important that adults nearing middle age take steps to prevent diseases and loss of physical function. To prolong healthy life expectancy and maintain national health, Taiwan’s health agency promotes adult preventive health services and physical checkup services for middle-aged and elderly individuals.

Aging is a natural process that inevitably affects physical function. The common body changes in aging are organ function decline or failure, bone loss, loss of muscle mass and strength, and body fat increases [3]. In turn, these changes increase the in-cidence of osteoporosis, sarcopenia, cardiovascular diseases, and other chronic dis-eases [4, 5]. Moreover, the infiltration of fat into the bone and muscle promotes the development of osteosarcopenic adiposity (OSA) and sarcopenia adiposity, diseases that cause a decline in endurance and body organ function, thereby increasing individuals’ risk of experiencing fatigue, falls, fractures, and death [6–8].

Recently, the term OSA was coined to describe a condition consisting of the sim-ultaneous occurrence of three conditions: osteopenia (loss of bone mass), sarcopenia (loss of muscle mass and strength), and adiposity (increase in body fat). For the elderly population, the infiltration of fat into the bone and muscle accompanied by metabo-lism dysfunction can lead to the decline in physical function that hinders independent living, which ultimately reduces the quality of life of an individual. Moreover, endo-crine imbalances and stem cell damage caused by cancer and diabetes can also in-crease an individual’s risk of developing OSA [9, 10]. Studies indicate that OSA is a silent epidemic that can eventually manifest through debilitating health complica-tions [10, 11].

Previous studies have found a positive relationship between metabolic syndrome and the individual conditions of sarcopenia [12], osteoporosis [13], and adiposity [14], which means that there is high risk for those who are diagnosed with metabolic syndrome to have sarcopenia, osteoporosis, and adiposity. However, to date, few studies have focused on the emerging integrated syndrome of OSA. Therefore, this study aimed to help fill this research gap by investigating the relationship between metabolic syndrome and OSA in the middle-aged and elderly populations.

## Methods

### Population and setting

This cross-sectional study used data from a sample of individuals whose information was included in the Eonway Health Management Center database, which was collected in 2016–2018. These data were obtained from physical examinations and questionnaire responses. The study subjects were all Taiwanese and above 50 years of age from urban areas; they had undergone a self-financed health checkup and had completed fitness exam questionnaire items. All records related to the physical examinations and questionnaire were collected by trained staff in the center and supervised by the in-charge physicians. All laboratory tests were measured in one laboratory. For individuals who had multiple records in the database from different years, only the latest record was utilized in the study. After the application of the selection criteria, 2991 cases remained and were used for study analysis (excluding 3251 cases which were younger than 50 years old, or those that had incomplete information either in survey items or in physical examination data). This study received approval for exempt review from the National Taiwan University (#202007EM017).

### Osteosarcopenic adiposity

The included subjects were divided into four groups according to the severity of OSA to explore its relationship with metabolic syndrome. The four groups were the controls (OSA = 0, without osteosarcopenic adiposity), those with either adiposity, sarcopenia, or osteoporosis (OSA = 1), those with any two of adiposity, sarcopenia, or osteoporosis (OSA = 2), and those with all the conditions, i.e., adiposity, sarcopenia, and osteoporosis (OSA = 3).

### Measurements

To be diagnosed with metabolic syndrome, an individual must meet the defined thresholds in three of the following five components: waist circumference, blood pressure, fasting blood glucose, fasting triglycerides, and high-density lipoprotein (HDL) according to the Ministry of Health and Welfare in Taiwan [15]. Following are the comprehensive five components criteria to define metabolic syndrome. Waist circumference must be ≧90 cm (35 in) in males or ≧80 cm (31 in) in females. For blood pressure, the individual must have a systolic blood pressure, SBP ≧130 mmHg and a diastolic blood pressure, DBP ≧85 mmHg or must be taking *antihypertensive agents.* For fasting blood glucose, the individual must be at ≧100 mg/dL or taking anti-diabetic agents. For fasting triglycerides, an individual must be at ≧150 mg/dL or taking hypolipidemic agents. Finally, HDL must be < 40 mg/dL in males or < 50 mg/dL in females.

For this study, osteopenia was defined by bone densitometry T-score (a comparison of a patient’s bone density with that of a healthy 30-year-old person of the same sex) of <− 1.0. WHO defines osteopenia as a T-score of − 1.0 to − 2.5 [16, 17]. Hospital employed dual-energy X-ray absorptiometry (DXA)—performed by trained registered nurses in the clinical setting—was used to collect data for diagnosing osteopenia. *The Asian Working Group for Sarcopenia (AWGS)* defines sarcopenia as a grip strength of < 18 kg in females and < 28 kg in males [18]. The hospital used a TKK-5401 machine operated by trained coaches to collect data for diagnosing sarcopenia. Adiposity was defined using the WHO standard of a body fat percentage of > 25% in males or > 35% in females [19].

### Covariates

The self-report questionnaire collected the respondents’ data on sex, age, living status, employment, exercise (almost none, walking or strolling, moderate-intensity exercise), nutrition supplementation (Vitamins B and D and calcium tablets), health behavior (smoking), and disease history (hypertension, hyperlipidemia, diabetes, and cardiovascular diseases). It should be noted that for the exercise, the questionnaire has provided some examples in the three levels for participants to choose their suitable situations The Cronbach’s alpha coefficient for all items on the survey was over 0.7.

### Statistical analyses

After displaying the distribution of demographic information, we investigated the relationship between OSA and the determining factors via chi-squared analysis. Multinomial logistic regression analysis was then performed to present OSA risk in individuals with metabolic syndrome after adjustment with other covariates. SPSS 20.0 (SPSS, Inc., Chicago, IL) was used to conduct all statistical analyses. The significance level was set to.05.

## Results

We presented the sample’s basic demographic information with distribution of the response items in Table 1. Of the total sample (2991), 1733 respondents were males (57.9%), with an average age of 58.55 ± 6.41 years. Regarding lifestyle measures, 2784 (93.1%) were living with family, 1657 (57.3%) were working full time, and 1206(41.1%) walked or participated in moderate-intensity exercise. With regard to supplementation, most of the respondents indicated “none” for Vitamin B (2,210, 73.9%), Vitamin D (2,789 93.2%), and calcium tablets (2562, 85.7%). For smoking, majority (2385, 80.3%) reported being non-smokers. For disease history, most of the respondents did not have hypertension (2221, 74.3%), hyperlipidemia (2503, 83.7%), diabetes (1645, 88.4%), or cardiovascular diseases (2854, 95.4%). Finally, 2319 (77.5%) respondents did not have metabolic syndrome.

**Table 1:** The basic characteristics with the distribution of response items among different OSA groups

	total	OSA = 0	OSA = 1	OSA = 2	OSA = 3	
variables	n(%)	n(%)	n(%)	n(%)	n(%)	*p*
Age	58.55 ± 6.41	55.63 ± 5.3	57.88 ± 5.89	59.63 ± 6.43	64.72 ± 8.02	< 0.001
Sex Male	1733(57.9)	193(11.1)	799(46.1)	668(38.5.1)	73(4.2)	< 0.001
Female	1258(42.1)	176(14)	624(49.6)	390(31)	68(5.4)	
Living status Alone	207(6.9)	26(12.6)	89(43)	81(39.1)	11(5.3)	0.563
With families	2784(93.1)	343(12.3)	1334(47.9)	977(35.1)	130(4.7)	
Employment Retired	847(29.3)	81(9.6)	378(44.6)	331(39.1)	57(6.7)	0.001
Part-time	390(13.5)	52(13.3)	187(47.9)	137(35.1)	14(3.6)	
Full-time	1657(57.3)	220(13.3)	815(49.2)	553(33.4)	69(4.2)	
Exercise almost none	600(20.4)	57(9.5)	266(44.3)	237(39.5)	40(6.7)	< 0.001
walking or strolling	1131(38.5)	110(9.7)	517(45.7)	447(39.5)	57(5)	
MI^a^ exercise	1206(41.1)	195(16.2)	614(50.9)	355(29.4)	42(3.5)	
Vitamin B No	2210(73.9)	278(12.6)	1026(46.4)	799(36.2)	107(4.8)	0.211
Yes	781(26.1)	91(11.7)	397(50.8)	259(33.2)	34(4.4)	
Vitamin D No	2789(93.2)	341(12.2)	1313(47.1)	1003(36)	132(4.7)	0.084
Yes	202(6.8)	28(13.9)	110(54.5)	55(27.2)	9(4.5)	
calcium tablets No	2562(85.7)	322(12.6)	1211(47.3)	908(35.4)	121(4.7)	0.763
Yes	429(14.3)	47(11)	212(49.4)	150(35)	20(4.7)	
Smoking No	2385(80.3)	288(12.1)	1139(47.8)	844(35.4)	114(4.8)	0.723
Yes	585(19.7)	79(13.5)	274(46.8)	208(35.6)	24(4.1)	
Hypertension No	2221(74.3)	307(13.8)	1091(49.1)	735(33.1)	88(4)	< 0.001
Yes	770(25.7)	62(8.1)	332(43.1)	323(41.9)	53(6.9)	
Hyperlipidemia No	2503(83.7)	320(12.8)	1214(48.5)	852(34)	117(4.7)	0.005
Yes	488(16.3)	49(10)	209(42.8)	206(42.2)	24(4.9)	
Diabetes No	2645(88.4)	338(12.8)	1265(47.8)	922(34.9)	120(4.5)	0.067
Yes	346(11.6)	31(9)	158(45.7)	136(39.3)	21(6.1)	
CV diseases No	2854(95.4)	359(12.6)	1370(48)	997(34.9)	128(4.5)	0.001
Yes	137(4.6)	10(7.3)	53(38.7)	61(44.5)	13(9.5)	
metabolic syndrome No	2319(77.5)	322(13.9)	1159(50)	737(31.8)	101(4.4)	< 0.001
Yes	672(22.5)	47(7)	264(39.3)	321(47.8)	40(6)	

CV: cardiovascular

OSA: osteosarcopenic adiposity

A: MI: moderate -intensity

The relationship between OSA and metabolic syndrome—determined via multinomial logistic regression—is presented in Table 2. After adjusting for sex, age, lifestyle, supplementation, smoking and disease history, the analysis revealed that the subjects with metabolic syndrome had an increased likelihood of having different severity levels of OSA (1, 2, and 3) with an odds ratio (OR) of 1.4 (95% CI = 0.99–1.99, *P* = 0.059), 2.49 (95% CI = 1.74–3.56, *P* < 0.001), and 2.57 (95% CI = 1.52–4.34, *P* < 0.001), respectively. After adjustment, the results demonstrated a positive relationship between the metabolic syndrome and OSA risk.

**Table 2:** the summary information in the relationship between metabolic syndrome and OSA from Multinomial logistic regression

metabolic syndrome (reference: No)		OR (95% CI)		Adjusted OR (95% CI)	
		OR(95%CI)	*p*	Adjusted OR(95%CI)	*p*
1 vs 0	Yes	1.56(1.12–2.18)	.009	1.4(0.99–1.99)	0.059
2 vs 0	Yes	2.98(2.14–4.16)	<.001	2.49(1.74–3.56)	<.001
3 vs 0	Yes	2.71(1.68–4.37)	<.001	2.57(1.52–4.34)	<.001

OSA: osteosarcopenic adiposity

OSA = 1: osteopenic or sarcopenic or adiposity

OSA = 2: osteosarcopenia or sarcopenic adiposity or osteopenic adiposity

OSA = 3: osteosarcopenic adiposity

More details from the multinomial logistic regression are presented in Table 3. The analysis indicated that age is not only do elderly individuals have an increased risk of OSA but also increment with the severity of OSA (from 1 to 3) with OR: 1.08 (95% CI = 1.05–1.11, *P* < 0.001), 1.13 (95% CI = 1.1–1.16, *P* < 0.001), and 1.26 (95% CI = 1.22–1.31, *P* < 0.001), respectively. With regard to exercise, in OSA = 1 group, the respondents engaging in moderate-intensity exercise (those who reported either “walking or strolling” or “almost none”) had an increased risk of OSA compared with those engaging in moderate-intensity exercise with OR: 1.49 (95% CI = 1.13–1.95, *P* = 0.004) and 1.64 (95% CI = 1.16–2.33, *P* = 0.005), respectively. Similar findings were observed in the OSA = 2 and OSA = 3 groups.

**Table 3:** the relationship between metabolic syndrome and OSA from Multinomial logistic regression

	1 VS 0		2 VS 0		3 VS 0	
Variables	AOR(95%CI)	***p***	AOR(95%CI)	*p*	AOR(95%CI)	*p*
Age	1.08(1.05–1.11)	<.001	1.13(1.1–1.16)	<.001	1.26(1.22–1.31)	<.001
Sex (ref: female)						
male	1.17(0.88–1.55)	.282	1.67(1.24–2.26)	.001	0.76(0.46–1.24)	.264
Living status (ref: with families)						
alone	0.9(0.56–1.45)	.666	1.15(0.7–1.9)	.576	0.84(0.36–1.92)	.673
Employment (ref: full-time)						
Part-time	0.82(0.57–1.17)	.271	0.8(0.55–1.18)	.263	0.42(0.21–0.85)	.016
Retired	0.98(0.71–1.35)	.882	1.17(0.83–1.64)	.375	0.64(0.38–1.09)	.102
Exercise (ref.: moderate -intensity exercise)						
walking or strolling	1.49(1.13–1.95)	.004	2.21(1.65–2.95)	<.001	2.22(1.35–3.65)	.002
almost none	1.64(1.16–2.33)	.005	2.69(1.86–3.88)	<.001	3.98(2.23–7.08)	<.001
Vitamin B (ref: Yes)						
No	0.83(0.62–1.1)	.186	0.94(0.69–1.27)	.678	1(0.6–1.65)	.987
Vitamin D (ref: Yes)						
No	1.19(0.75–1.87)	.466	1.64(0.98–2.74)	.06	1.37(0.59–3.22)	.465
calcium tablets (ref: Yes)						
No	0.91(0.63–1.29)	.584	0.95(0.65–1.38)	.786	1.15(0.62–2.14)	.65
Smoking (ref: Yes)						
No	1.2(0.87–1.65)	.257	1.37(0.98–1.92)	.068	1.02(0.56–1.85)	.944
Hypertension (ref: Yes)						
No	0.82(0.59–1.14)	.238	0.71(0.51–1)	.053	0.67(0.4–1.1)	.116
Hyperlipidemia (ref: Yes)						
No	1.05(0.72–1.51)	.813	0.9(0.61–1.31)	.579	1.16(0.63–2.16)	.629
Diabetes (ref: Yes)						
No	1.04(0.67–1.62)	.861	1.28(0.81–2.03)	.286	1.11(0.57–2.15)	.754
CV disease (ref: Yes)						
No	1.02(0.5–2.08)	.947	0.81(0.39–1.65)	.554	0.55(0.21–1.41)	.212
metabolic syndrome (ref: Yes)						
No	1.4(0.99–2)	.059	2.49(1.74–3.56)	<.001	2.57(1.52–4.34)	<.001

OSA = 1: osteopenic or sarcopenic or adiposity

OSA = 2: osteosarcopenia or sarcopenic adiposity or osteopenic adiposity

OSA = 3: osteosarcopenic adiposity

## Discussion

In this study, metabolic syndrome was found to be a significant factor among the four OSA groups after adjusting for other variables. This positive relationship between metabolic syndrome and OSA is in line with previous studies indicating that metabolic syndrome contributed not only to OSA but also to osteoporosis, sarcopenia, and adiposity [20–22]. Another factors associated with OSA are age, sex, job type and regular exercise. Aging in itself remains an important factor for the development of osteoporosis, sarcopenia, and adiposity, with other studies indicating that individuals above 50 years old have an increased OSA risk compared with younger individuals [23, 24].

Another factor, gender, has reported as a significant factors in previous studies, which reflect the differences in the physiques between males and females; females may lose up to 10% of their bone mass in the first five years after menopause and be led towards high risk of being diagnosed with osteoporosis [25, 26]. This study also reveals similar findings; for example, the T-score for females and males were − 1.68 ± 1.31 and − 1.35 ± 0.97, respectively. The greater the negative value, the lower the bone mass density; this is interpreted based on the guidelines proposed by World Health Organization (WHO). In addition, we found males had 1.67 times higher for OSA than females in OSA = 2, while the opposite result in females was observed with 24% reduced risk in OSA = 3. The possible explanation is that there is no difference in body fat and muscle power between males and females during the aging process, while bone mass loss occurs in females due to menopause, increasing the risk of OSA.

Concerning employment type, individuals who are working part-time or are retired had a lower likelihood of having OSA (22–74%) compared with those working full time. This finding is not consistent with those other studies indicating that retired people experience diminishing muscle strength [27]. In addition, blue-collar workers also have an increased risk of OSA and mortality, possibly due to this group’s tendency to make unhealthy life choices [28]. However, despite the findings of the current study, confirming the association between employment type and OSA risk requires more research. In other words, further studies are needed to investigate how employment type and retirement duration affect OSA risk.

In the current study, although limited information from exercise items, the group with the least exercise had two to three times the risk of OSA. These findings help confirm previous findings suggesting that—compared with those with a sedentary lifestyle or moderate-intensity exercise participation—those with regular or high-intensity exercise have a reduced OSA risk [29–31]. Those studies also confirm that exercise can help reduce body fat percentage and improve muscle mass and power, outcomes that also can help reduce the risk of OSA and other age-related diseases.

Metabolic syndrome, age, sex, job types, and exercise are related to the risk of OSA. Although age and sex inherited most influenced risk, people improve muscle power and bone mass with reduced body fat from regular exercise and changing diet habits. These efforts can maintain the certain level of quality of life, reducing the risk of autonomous life function and ability loss.

Healthcare providers should remind individuals at high risk for OSA to exercise regularly (to prevent bone and muscle loss), reduce their intake of foods high in saturated fats (e.g., red meat, French fries, and bacon), and consume more protein and Vitamin D [32]. In addition, we recommend weight-bearing exercises (such as speed walking, swimming, and running) to increase quadricep strength as well as regular exercise (yoga, strolling, and Pilates) to prevent OSA. These suggestions are effective for reducing OSA risk and can even reduce body fat, improve fitness, and reduce the incidence of falls and bone fractures [33–36]. Therefore, we encourage early investigation of and intervention for OSA among individuals who have abnormal findings in their regular health examinations.

### Limitations

The study has several limitations that must be noted. First, because this is a cross-sectional study, it is difficult to determine the relative causality of each factor. Instead, the findings present the relationship between metabolic syndrome and OSA risk. Second, because the study group comes from a single healthcare examination center in one hospital, we must consider selection bias, especially the distribution of demographic characteristics, such as employment type and lifestyle. For this reason, these results may not be generalizable to other elderly populations in different regions. Third, because the study group consists of individuals who used self-pay health examination services, their health status and physical condition may be better than their counterparts, especially in basic physical performance and self-management abilities. Therefore, we did not discuss those who had restricted physical function or health status. Fourth, while most previous studies used muscle mass and strength to define OSA, we used muscle strength only to select OSA cases (according to Asian (2020) AWGS2020). Using this strategy may have resulted in an underestimation of OSA risk. Fifth, with regard to job type, previous studies have demonstrated that the consumption of different job types would be associated with the risk of OSA. Therefore, further study is required to elucidate the possible effects of alcohol type and quantity on various health measures. With regard to employment type, we only used three designations (full time, part-time, or retired), ignoring other information, such as job type. Contrarily, because other studies have indicated that different types of jobs affect OSA risk, this topic requires further study. Finally, most studies have pointed out the diet habit with the association of OSA; however, we did not have sufficient information from the survey items, which should be considered in further studies.

## Conclusion

For the current study, we analyzed health examination data to learn about the relationship between OSA and metabolic syndrome, especially in elderly, people lacking exercise habits, or females. These results can help promote early diagnosis and treatment of OSA in clinical settings, particularly among aging individuals with abnormal physical function, the group with the highest OSA incidence.

## Data Availability

Restrictions apply to the availability of these data. Data was obtained from Eonway Health Management Center and are available from Yu-Hsiang Su with the permission of Eonway Health Management Center.

## Appendix

**Table:**

	Male	Female
**Variables**	Mean ± SD	Mean ± SD
Age	58.76 ± 6.53	58.25 ± 6.23
Grip strength	38.47 ± 6.75	22.79 ± 4.2
Body fat	26.31 ± 5.44	33.47 ± 5.99
BMD	−1.35 ± 0.97	−1.68 ± 1.31
BMI	25.44 ± 3.04	23.28 ± 3.24
SBP	127.1 ± 13.66	123.05 ± 16.05
DBP	76.96 ± 9.87	70.94 ± 10.58
WA	90.85 ± 8.27	80.22 ± 8.18
GLU-AC	106.51 ± 25.89	100.22 ± 21.86
TG	136.47 ± 91.51	105.04 ± 62.51
HDL-C	48.32 ± 11.45	61.31 ± 15.23

## Abbreviations

CI: confidence interval
DBP: diastolic blood pressure
DXA: dual-energy X-ray absorptiometry
ERI: effort-reward imbalance
AWGS: Asian Working Group for Sarcopenia
HDL: high-density lipoprotein
NCDs: noncommunicable diseases
OR: odds ratio
OSA: osteosarcopenic adiposity
SBP: systolic blood pressure
WHO: World Health Organization
